# Licensing of Orphan Medicinal Products—Use of Real-World Data and Other External Data on Efficacy Aspects in Marketing Authorization Applications Concluded at the European Medicines Agency Between 2019 and 2021

**DOI:** 10.3389/fphar.2022.920336

**Published:** 2022-08-11

**Authors:** Frauke Naumann-Winter, Franziska Wolter, Ulrike Hermes, Eva Malikova, Nils Lilienthal, Tania Meier, Maria Elisabeth Kalland, Armando Magrelli

**Affiliations:** ^1^ Federal Institute for Drugs and Medical Devices, Bonn, Germany; ^2^ Department of Pharmacology and Toxicology, Faculty of Pharmacy, Comenius University, Bratislava, Slovakia; ^3^ State Institute for Drug Control, Bratislava, Slovakia; ^4^ Norwegian Medicines Agency, Oslo, Norway; ^5^ National Center for Drug Research and Evaluation, Istituto Superiore di Sanità, Rome, Italy

**Keywords:** real-world data, drug development, orphan medicinal product, orphan drugs, efficacy, marketing authorization application

## Abstract

**Background:** Reference to so-called real-world data is more often made in marketing authorization applications for medicines intended to diagnose, prevent or treat rare diseases compared to more common diseases. We provide granularity on the type and aim of any external data on efficacy aspects from both real-world data sources and external trial data as discussed in regulatory submissions of orphan designated medicinal products in the EU. By quantifying the contribution of external data according to various regulatory characteristics, we aimed at identifying specific opportunities for external data in the field of orphan conditions.

**Methods:** Information on external data in regulatory documents covering 72 orphan designations was extracted. Our sample comprised public assessment reports for approved, refused, or withdrawn applications concluded from 2019–2021 at the European Medicines Agency. Products with an active orphan designation at the time of submission were scrutinized regarding the role of external data on efficacy aspects in the context of marketing authorization applications, or on the criterion of “significant benefit” for the confirmation of the orphan designation at the time of licensing. The reports allowed a broad distinction between clinical development, regulatory decision making, and intended post-approval data collection. We defined three categories of external data, administrative data, structured clinical data, and external trial data (from clinical trials not sponsored by the applicant), and noted whether external data concerned the therapeutic context of the disease or the product under review.

**Results:** While reference to external data with respect to efficacy aspects was included in 63% of the approved medicinal products in the field of rare diseases, 37% of marketing authorization applications were exclusively based on the dedicated clinical development plan for the product under review. Purely administrative data did not play any role in our sample of reports, but clinical data collected in a structured manner (from routine care or clinical research) were often used to inform on the trial design. Two additional recurrent themes for the use of external data were the contextualization of results, especially to confirm the orphan designation at the time of licensing, and reassurance of a large difference in treatment effect size or consistency of effects observed in clinical trials and practice. External data on the product under review were restricted to either active substances already belonging to the standard of care even before authorization or to compassionate use schemes. Furthermore, external data were considered pivotal for marketing authorization only exceptionally and only for active substances already in use within the specific therapeutic indication. Applications for the rarest conditions and those without authorized treatment alternatives were especially prominent with respect to the use of external data from real-world data sources both in the pre- and post-approval setting.

**Conclusion:** Specific opportunities for external data in the setting of marketing authorizations in the field of rare diseases were identified. Ongoing initiatives of fostering systematic data collection are promising steps for a more efficient medicinal product development in the field of rare diseases.

## Introduction

Recent reviews reported that evidentiary support from the so-called real-world data (RWD) is more prevalent among marketing authorization applications (MAAs) for orphan medicinal products (OMPs) compared with MAAs for products treating common diseases (Eskola et al., 2022; Flynn et al., 2022; Mahendraratnam et al., 2022; Purpura et al., 2022). Indeed, in the setting of rare diseases, and especially along a protracted disease course, either patients or clinical events—or both—may be scarce (EURORDIS, 2022). This may raise the challenge of comprehensively collecting data within a dedicated clinical development program and underlines the importance of external data to complement such developments (Goring et al., 2019; Tambuyzer et al., 2020). External information has traditionally been reported to inform clinical development (e.g., with respect to adequate trial design or sample size), and increasingly to provide context (e.g., external control groups), or to contribute to regulatory decision making (Cave et al., 2019; Franklin et al., 2019; Dagenais et al., 2022).

Many different definitions of RWD sources (and their derived evidence) are used in the scientific or regulatory literature (Concato and Corrigan-Curay, 2022; BDSG, 2021). At large, RWD refers to data obtained through multiple sources, which are related to patient health status or delivery of health care and medical clinical practice, but not to data derived from “traditional” prospective, potentially randomized, clinical trials (RCT). A wide spectrum of data sources (or study designs) is collectively referred to as representing the real world from purely administrative to prospective observational research (Makady et al., 2017). These types of data strongly differ with respect to the depth of clinical information that is collected, and, depending on the reporting format, provided for assessment (Karpen et al., 2021). Administrative data are restricted to diagnostic codes that—by a combination of codes and their chronological order—may be used to describe the (long-term) consequences of specific treatment patterns. However, if a disease is heterogeneous, such as many rare diseases, more information is typically required to understand the specific course of the disease and the potential impact of prognostic factors or treatments. Such insight can usually only be gained from data that are entered or curated by health care professionals, and typically requires a protocol-guided assessment to avoid bias and confounding.

**FIGURE 1:**
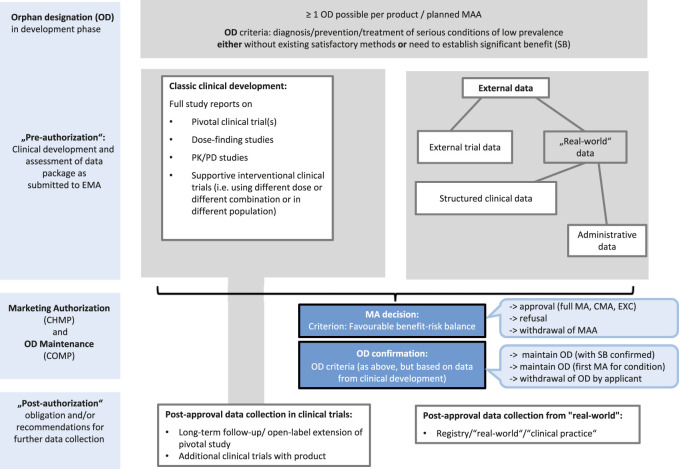
Clinical development and regulatory processes for products with orphan designation. CHMP: Committee for Medicinal Products for Human Use; CMA: conditional MA, COMP: Committee for Orphan Medicinal Products; EMA: European Medicines Agency; EXC: MA under exceptional circumstances; MA(A): marketing authorization (application); PD: pharmacodynamic; PK: pharmacokinetic; OD: orphan designation; SB: significant benefit.

In our understanding, both observational research and clinical trials are performed with real patients in the real world under conditions which are—more or less—controlled by the selected inclusion and exclusion criteria and the specific study protocol. For our analysis of a broad sample of recent orphan drug developments, we, therefore, included all explicit references to data outside the standard clinical development plan for the product under review as external data (Figure 1). In view that only 5% of the 5,000–8,000 rare diseases that are presently recognized (Haendel et al., 2020) have a treatment available, orphan designated medicinal products (MPs) correspond to an area where an increase in the efficiency of clinical development or regulatory decision making is especially needed (European Commission, 2020).

To receive MA, a product needs to demonstrate a favorable benefit-risk balance based on the data provided on the clinical development as assessed by the Committee for Human Medicinal Products (CHMP). The benefit-risk balance may need to be monitored or confirmed even after MA in the post-approval phase when there is uncertainty on specific aspects as identified during the evaluation of the MAA. At the time of MA, orphan designated MPs also need to confirm the orphan criteria with the Committee for Orphan Medicinal Products (COMP) (European Parliament and of the Council, 2000). Prevalence is required to be below five in 10,000 persons in the EU and “significant benefit” (SB) may need to be shown if other “satisfactory methods” already exist for the targeted therapeutic indication (Fregonese et al., 2018; Sheean et al., 2021). The less a condition is understood, the more challenging drug development may be. The lack of understanding of a condition may be linked to its rarity (Tambuyzer et al., 2020). The category of orphan designated MPs not requiring a discussion of the SB at the time of MA may, therefore, highlight an area of especially high unmet medical need as indicated by a complete lack of prior successful developments of therapeutic approaches.

In our regulatory landscape analysis, we provide granularity on the use of external data for supporting efficacy aspects in orphan MAAs. We focus on clarifying its contribution to efficacy aspects only, since the contribution of routinely collected data on safety is already more established. Our primary objective is to review and quantify the contribution of external data in the course of three regulatory phases: first, we take note of any impact of external data on the clinical development or their assessment in European public assessment reports (EPARs) by the CHMP. Second, we highlight whether questions with respect to efficacy aspects remain even after approval and categorize the data sources recommended for post-approval data collection into RWD or clinical trials. Third, we elucidate the role of external data to justify the SB at the time of MA and its assessment by the COMP. Furthermore, we characterize our sample of orphan MAAs by referring to regulatory information derived from reports by two regulatory committees at the European Medicines Agency (EMA). Thereby, we aim at identifying specific opportunities for external data when submitted to contribute to efficacy assessment across the regulatory life-cycle of MPs in the setting of rare diseases.

## Methods

Public documents from the EMA website were screened for references to external data sources (Table 1 and Figure 1) regarding the information on 1) efficacy aspects in EPARs on both the pre-approval and post-approval phases and 2) fulfillment of the criterion of SB in Orphan Maintenance Assessment Reports (OMARs). Annex II D of the approved product’s information describes the legally binding post-approval data collection, while further discussion on the usefulness of the post-approval data collection may also be mentioned in the discussion of the benefit-risk balance in the EPAR.

**TABLE 1:** Definition of classification of external data described in EPARs, OMARs, and Annex II D.

External trial data (ETD)	Real-world data	
External interventional trial data	Structured clinical data collection including observational research (SCD)	Administrative data
• Published clinical trials	• Retrospective review/clinical experience (may include chart review)	
• Meta-analysis	• Prospective data collection	• Claims data
	• Data systematically collected by patient organizations	• Prescription data
	• Cohort study	• Drug dispensing data
• Individual patient-level data from clinical trials	• Early access or compassionate use programme	
	• Natural history	
	• Registry (study)	
	• (Individual patient-level data)	
**Not considered as external data:** clinical trials with products as applied for, dose-finding, phase I–III (including follow-up after interim analysis), open-label follow-up of clinical trials, surveys not based on individual patients, pharmacokinetic or pharmacodynamic studies, and drug–drug interaction studies.		

All MAAs for initial MAs finalized between 2019 and 2021 at the EMA were identified on its website and the community register of OMPs and cross-checked with internal information. MAAs with an active orphan designation (OD) at the time of submission were selected for analysis. Since one active substance may have more than one OD, the data were analyzed per OD for most of the applications. Three active substances had been designated for several orphan conditions before the submission of the MAA, but the discussions within the EPAR and OMAR at the time of MA were identical for all ODs within the resulting MA. To avoid overestimating the impact of the specific characteristics of the associated dossiers, these ODs were counted only as one OD.

To assess which types of external data (either so-called RWD or data from clinical trials) had been submitted for a specific product, three major categories of external data were defined (Table 1 and Figure 1). We distinguished administrative data, structured clinical data (SCD), and external trial data (ETD). Administrative data refer to data generated to support billing and supply and are expected to have the least detail with respect to clinical information on a specific patient along the disease course. Clinical information collected by a health care professional to reflect the individual patient’s disease trajectory, for example, health records or a registry was classified as SCD, a broad spectrum between routine clinical documentation and observational research. This category was also used if pooled data from several external data sources were described and for data from early access/compassionate use programs.

Finally, ETD were defined as being derived from “re-used” clinical trials as published clinical trials, meta-analyses of clinical trials, or individual patient-level data from clinical trials studying different MPs from the one under review. Although clinical trial data themselves are usually excluded from the definition of RWD, they were included in our analysis since both RWD and ETD may be used for similar reasons. The ETD category was also established to complete the spectrum of the potentially informative value of external data sources from administrative to research purposes. Furthermore, the format in which the ETD were available, being either aggregate or individual patient-level data, was collected (if reported) since this may restrict its informative value. If more than one external data source of at least two different categories were identified in a report, both categories were extracted and used for further analysis.

Information on the ATC code, type of MA, design of the pivotal study(ies), and orphan status at the time of EC approval was extracted from the EPAR of approved, refused, or withdrawn MAAs. Disease prevalence was either extracted from the OMAR or determined based on present knowledge. Only pivotal, supportive, or otherwise explicitly described or tabulated data from regulatory documents were considered relevant for our analysis, whereas introductory references to the therapeutic indications were not classified as external data.

It was determined whether external data were focused on the product under review or if they provided information on the therapeutic context (e.g., natural history study). Moreover, the intended use of the external data by the applicants or the regulators was noted as descriptive, informing, or formal. To be categorized as descriptive, the external data sources were reported, but no further statistical analyses were performed. Data were considered informing if external data were used to inform on the clinical development under review such as on the choice of endpoints, sample size, and non-inferiority margins. External data were also considered informing when regulators made reference to them to justify a specific wording of the indication. The formal use approach was noted if new statistical analyses or presentation of data, presumably not planned at the time of the original data collection, were performed.

Furthermore, we extracted several characteristics systematically available for all ODs and quantified the contribution of external data across these subgroups. Although withdrawal assessment reports and refusal reports were included for the overall analysis, the analyses according to the basic regulatory characteristics were only performed for those products which obtained MA (Table 2). When less than four ODs shared a specific characteristic (5% of EPARs, 7% of OMARs), this subgroup was considered too small for meaningful analysis and was also left out of the figures (e.g., RCT + SAT n = 2, EXC + SB n = 2).

**TABLE 2:** Characteristics collected for the sample of ODs included in authorized products.

Characteristics collected	All authorized	MA + SB	MA − SB
	(N = 60 OD) n (%)	(N = 46 OD) n (%)	(N = 13 OD) n (%)
**Type of authorization**			
Full authorization	39 (**65**)	32 (**70**)	7 (**54**)
Conditional MA	17 (**28**)	12 (**26**)	4 (**31**)
MA under exceptional circumstances	4 (**7**)	2 (**4**)	2 (**15**)
**ATC class**			
A	8 (**13**)	4 (**9**)	4 (**31**)
B	7 (**12**)	7 (**15**)	0 (**0**)
C, P, R	3 (**5**)	2 (**4**)	1 (**8**)
H	3 (**5**)	3 (**7**)	0 (**0**)
J	4 (**7**)	3 (**7**)	1 (**8**)
L	27 (**45**)	22 (**48**)	4 (**31**)
M	3 (**5**)	2 (**4**)	1 (**8**)
N	5 (**8**)	3 (**7**)	2 (**15**)
**Active substance status**			
New active substance	51 (**85**)^b^	37 (**80**)	13 (**100**)
Known active substance	9 (**15**)	9 (**20**)	0 (**0**)
**Design of the pivotal trial**			
SAT	24 (**40**)^a^	16 (**35**)	7 (**54**)
RCT	34 (**57**)	28 (**61**)	6 (**46**)
Both RCT + SAT	2 (**3**)	2 (**4**)	0 (**0**)
**Prevalence (per 10,000)**			
<1	31 (**52**)	22 (**48**)	9 (**69**)
1 - <3	14 (**23**)	12 (**26**)	2 (**15**)
≥3	15 (**25**)	12 (**26**)	2 (**15**)
**“Significant benefit”**			
OD without SB	13 (**22**)	NA	13 (**100**)
OD with SB	47 (**78**)^c^	46 (**100**)	NA
**Orphan status beyond MA**			
Orphan status maintained	46 (**77**)	33 (**72**)	13 (**100**)
Orphan status withdrawn	14 (**23**)^c^	13 (**28**)	0 (**0**)

ATC: anatomical therapeutic chemical classification, MA: marketing authorization, OD: orphan designation, RCT: randomized clinical trial, SAT: single-arm trial, SB: significant benefit.

a One OD, with retrospective pivotal data was included in the SAT analysis set.

b One OD was classified as new active substance by the EMA, but is known and used outside the EU.

c Including one withdrawn OD without OMAR excluded for analysis in the dataset with n = 46 OMAR.

The sample of regulatory documents was divided among authors. All authors extracted data into excel spreadsheets with proposals for the classification of data in the aforementioned categories. All classified information was cross-checked twice to establish consistency. Disagreements were resolved by discussion between at least two authors or within the group.

## Results

### Characteristics of the Sample of MAAs Analyzed

**FIGURE 2:**
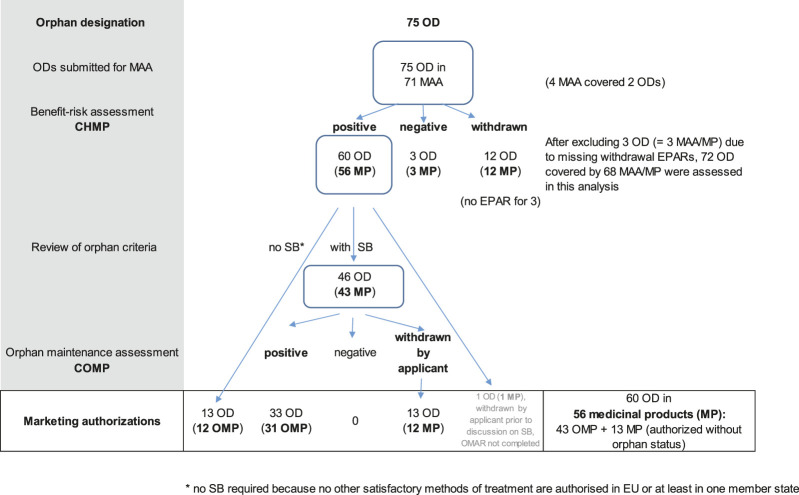
Flow-chart on the regulatory pathway of the sample of ODs assessed for the present analysis. CHMP: Committee for Medicinal Products for Human use; COMP: Committee for Orphan Medicinal products; EPAR: European public assessment report; MA(A): marketing authorization (application), MP: medicinal product; OD: orphan designation; OMAR: orphan maintenance report; OMP: orphan medicinal product; SB: significant benefit.

Overall, 71 MAAs including 75 ODs were finalized between January 2019 and December 2021 at the EMA. Four MAAs included more than one OD (Figure 2). Overall, 56 MPs covering 60 ODs were approved, 12 ODs in 12 MAAs were withdrawn by the applicant during the regulatory review, and three ODs in three MAAs were refused by the CHMP. For three withdrawn MAAs, EPARs were not yet published by the data cut-off (April 1, 2022) and these were therefore excluded from our analysis. In the following, all analyses are reported according to the number of ODs included in MAAs which were recommended for approval by the CHMP.

The majority of the ODs resulted in full approval (39 ODs), 17 ODs were approved as conditional MA (CMA) and four “under exceptional circumstances” (EXC) (Table 2). The sample of 60 ODs covered 10 different therapeutic areas, the majority of which was within the field of oncology (45%) followed by metabolic diseases (13%) and disorders of blood and blood-forming organs (12%). Most ODs concerned new active substances (n = 51; 85%), while nine ODs (15%) referred to substances known in the EU. One active substance was considered new for the European market but corresponded to standard of care outside of the EU. The trial submitted as pivotal in the MAA was designed as a RCT for more than half of the ODs (57%), while single-arm trials (SAT) had been submitted for 40% of the ODs. For two ODs (3%), both RCT and SAT data had been submitted.

Approximately half of the orphan conditions were very rare (prevalence <1 in 10,000; 52%), while the two other categories of prevalence (1 - <3 and 3 - <5 in 10,000) were almost equally represented in our sample. To benefit from the 10-years of market exclusivity of an OMP, 13 ODs (22%) needed to confirm the prevalence criterion only. The other 47 ODs (78%) were required to justify the criterion of SB for maintaining the OD beyond MA in view that satisfactory methods of treatment already existed. Of the latter, the COMP recommended maintenance of the OD for 33 ODs (77%) which were then authorized as 31 OMPs. The applicant formally withdrew 14 ODs before MA. Of these, 13 ODs were withdrawn by the applicant after a list of questions on SB had been issued by the COMP and were subsequently authorized, without an orphan status, as 12 MPs. One additional OD had been withdrawn before approval by the applicant at an earlier stage without prior submission of data and was, therefore, excluded from the analysis of OMARs. Overall, 46 ODs were maintained at the time of MA (with or without SB) and were subsequently authorized as 43 OMPs.

The overall distribution of these regulatory categories was similar in the sample of 60 ODs included in all authorized MPs compared with the subgroup of 46 ODs where the establishment of SB was required for confirmation of the OD (Table 2). Of note, 76% (13 of 17) of ODs included in CMAs also required the justification of SB for a subsequent discussion with the COMP. Half of the products for the treatment of metabolic diseases were developed for orphan conditions without available treatment options, while SB had to be established for most of the ODs of antineoplastic and immunomodulatory agents (ATC code: L) and all products targeting blood and blood-forming organs (ATC code: B). Only new active substances were studied for areas for which satisfactory methods were lacking (13 ODs), and all known active substances had to demonstrate SB. In the (small) group of ODs without the need for confirmation of SB, a higher proportion of pivotal studies was designed as SAT (7 of 13; 54% vs. 16 of 46, 35%) and the products were more often not fully authorized (6 of 13; 46% vs. 14 of 46, 30%).

### Structured Clinical Data More Common During Clinical Development

**FIGURE 3:**
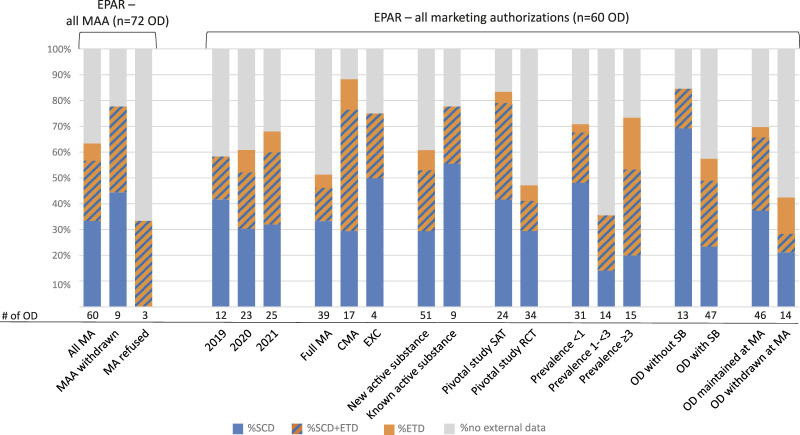
Types of external data submitted in the pre-authorization period. N = 72 (all EPARs) or n = 60 ODs (only EPARs on successfully authorized products), respectively, were searched for reference to external data and analyzed for the type of data. The numbers of subgroups are stated below the columns. The subgroup “SAT + RCT” was considered too small to be included in the figure (n = 2) but is tabulated in Supplementary Table S1. CMA: conditional marketing authorization; EPAR: European public assessment report; ETD: external trial data; EXC: marketing authorization under exceptional circumstances; MA (A): marketing authorization (application); OD: orphan designation; RCT: randomized controlled trial; SAT: single-arm trial; SB: significant benefit; SCD: structured clinical data.

Overall, in 64% of all 72 ODs included in our sample of MAAs, external data were mentioned in the efficacy sections of the EPAR, where 33% (24 of 72) of these comprised SCD, 6% (4 of 72) of ETD, and 25% (18 of 72) both (Supplementary Table S1). In MAAs which were withdrawn before the final opinion by the CHMP, external data were included more frequently (7/9; 78%) than in successful MAAs (38/60; 63%). No reference at all was made to administrative data, and overall, SCD were more commonly submitted with the pre-approval data package compared to ETD (Figure 3). There was no notable change with respect to the discussion of external data in EPARs during the three calendar years examined.

Of all authorized products (60 ODs), external data were most often submitted for MPs authorized as CMA (15 of 17; 88%) or EXC (3 of 4; 75%) as opposed to a full MA (20 of 39; 51%). Although SCD were overall more commonly submitted across all authorized products (33 of 60; 55%), the highest proportion of ETD (10 of 17; 59%) was found for CMA. For the few ODs approved in the setting of an EXC, SCD were submitted in three of four applications (75%).

Applications with a new active substance status had a lower frequency of reference to external data (31 of 51; 61%) than known substances (7 of 9; 78%). MAAs with pivotal data corresponding to SATs were also more frequently supported by external data than MAAs with pivotal data from RCTs (20 of 24, 83% vs. 16 of 34, 47%). For both study designs, SCD were submitted more frequently than ETD (SAT: 79% vs. 42%; RCT: 41% vs. 18%). While the proportion of reports referring to external data overall was comparable for the highest and lowest prevalence category (73% vs. 71%), SCD were found more often for MAs targeting the rarest diseases (21 of 31, 68%) than those targeting diseases with a prevalence between 3 and <5 in 10,000 (8 of 15, 53%). ETD, however, contributed much more to external trial data in the category of prevalence above 3 in 10,000 (8 of 15; 53%) than the lowest prevalence category (7 of 31, 23%). The highest proportion of SCD was found in MAs targeting orphan conditions without existing satisfactory methods (11 of 13, 85%), in which SCD contributed to a higher extent than to ODs targeting therapeutic areas for which the criterion of SB was mandatory (23 of 47, 49%). The inverse is observed for ETD which were submitted twice as often in the setting of ODs where the SB criterion had to be fulfilled (16 of 47; 34%) compared to those without the need to demonstrate SB (2 of 13; 15%). Finally, external data were more often submitted in regulatory procedures for which the orphan status was maintained than those where the applicant withdrew the OD before approval (70% vs. 43%).

### Real-World Data Collection After MA

The CHMP stated that further information on efficacy would be important even after approval and expected the final study report of the ongoing dedicated clinical studies for review at a later stage (33 of 60; 55%). Reference to a recommendation to collect data on efficacy aspects from the “real-world,” “routine clinical practice,” or a “registry” was found for 32% (19 of 60) of the ODs authorized. When this latter subgroup of ODs is characterized further (data not shown), the following features are most prominent: CMAs and EXC (10 of 19; 53%), pivotal studies designed as SAT (13 of 19; 68%), and the most pronounced rarity of the targeted diseases (14 of 19; 74%). Interestingly, 16 of these 19 ODs (84%) with RWD collection recommended beyond MA had already included external data in the pre-authorization data package (SCD 16 of 19; 84%; ETD 6 of 19; 32%; no external data 3 of 19 (16%)). In addition, if ODs of all authorized products are categorized according to the submission of external data in the pre-approval data package, 16 of 38 ODs with external data in the pre-approval data package (42%) were asked to continue RWD collection beyond MA, while only three of 22 ODs without external data in the pre-approval data package were recommended to collect RWD post-approval (14%). Data collection post-approval was most often recommended in the setting of an observational registry to be agreed upon with the regulators but no details on the exact study plan were available at the time of MA.

### External Data for Establishing “Significant Benefit”

**FIGURE 4:**
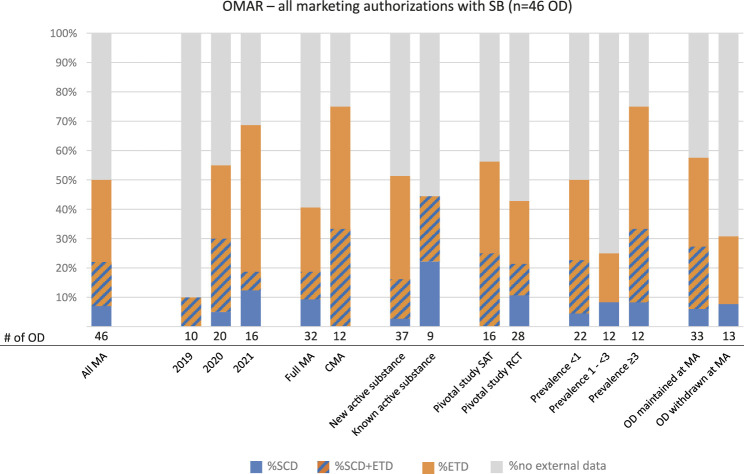
Types of external data submitted for the justification of “significant benefit”. N = 46 ODs (OMARs on successfully authorized products, which needed to comply with the “significant benefit” criterion) were searched for reference to external data and analyzed for the type of data. The numbers of subgroups are stated below the columns. The subgroups “SAT + RCT” (n = 2) and “MA under exceptional circumstances” (n = 2) were considered too small to be included in the figure but are tabulated in Supplementary Table S1. CMA: conditional marketing authorization; OMAR: orphan maintenance assessment report; ETD: external trial data; MA (A): marketing authorization (application); OD: orphan designation; RCT: randomized controlled trial; SAT: single-arm trial; SB: significant benefit; SCD: structured clinical data.

SB assessment for the maintenance of the OD beyond MA was required for 46 ODs (77%). Again, administrative data were not mentioned in any OMAR (Table 3). Contrary to the situation observed in EPARs, ETD were more often described than SCD (Figure 4). A total of 43% (20 of 46) referred to ETD whereas only 22% (10 of 46) of ODs included SCD. SCD were submitted mostly in the subgroup of known active substances (4 of 9, 44%), where conditions were closer to the orphan prevalence threshold (3 - <5 in 10,000) (4 of 12, 33%) and CMAs (4 of 12, 33%). ETD were mostly found in OMARs on products granted CMA (9 of 12, 75%) and conditions at the upper range of the prevalence spectrum eligible for OD (3 - <5 in 10,000) (8 of 12, 67%). For products with an RCT as the pivotal study, a high proportion of ETD was also submitted in the setting of the confirmation of ODs (9 of 28; 32%). Of note, fewer procedures for which the OD was withdrawn referred to external data compared to those which maintained the OD at the time of MA. A steady rise was observed during the three calendar years analyzed with 69% (11 of 16) of orphan maintenance procedures containing reference to external data in 2021 compared to only 10% in 2019.

**TABLE 3:** Object, source, and purpose of submission of the external data.

Type of report	EPAR		OMAR		Post-approval recommendations for RWD collection (n = 60 OD) n (%)	
	MA (n = 60 OD) n (%)		MA + SB (n = 46 OD) n (%)			
	Context^a^	Product^a^	Context^b^	Product^b^	Context	Product
**All MAs**						
Any external data	32 (**53**)	11 (**18**)	22 (**48**)	2 (**3**)^c^	8^d^ (**13**)	19 (**32**)
SCD	25 (**42**)	11 (**18**)	9 (**20**)	1 (**2**)^c^	8^d^ (**13**)	19 (**32**)
		(6 EAP; 6 AS^c^)				
ETD	17 (**28**)	3 (**5**)^c^	20 (**43**)	1 (**2**)^c^	—	—
Administrative data	0 (**0**)	0 (**0**)	0 (**0**)	0 (**0**)	0 (**0**)	0 (**0**)

AS: active substance, EAP: early access program, EPAR: European public assessment report, ETD; external trial data, MA: marketing authorization, OMAR: orphan maintenance assessment report, SB: significant benefit, SCD: structured clinical data.

a Or both (overlap in five ODs).

b Or both (overlap in 1 OD).

c Known active substances, or use outside of the EU.

d Complete overlap with the 19 ODs in the last column.

### External Data Mainly Used to Capture Context

During the pre-authorization phase and for the demonstration of SB, external data were more frequently focused on the clinical context than on the product under review (Table 3). The external data on the product described in the EPAR for the pre-authorization phase were either from a different, already authorized formulation of the identical active substance or from an early access/compassionate use program. For the post-authorization phase, the CHMP recommended collecting RWD on efficacy aspects of the authorized product in a real-world setting in 19 MA procedures (32%). Interestingly, the CHMP stated the importance of data collection also from untreated patients (natural history) or alternative treatments (e.g., allogeneic transplantation) or explicitly recommended analyses from disease registries for eight of these 19 ODs (42% or 13% of all ODs covered in MAs). All eight ODs concerned diseases of the category of the lowest prevalence but were otherwise not notably enriched for a specific design of the pivotal study, the requirement for SB, or the type of approval.

### Use of External Data

**FIGURE 5:**
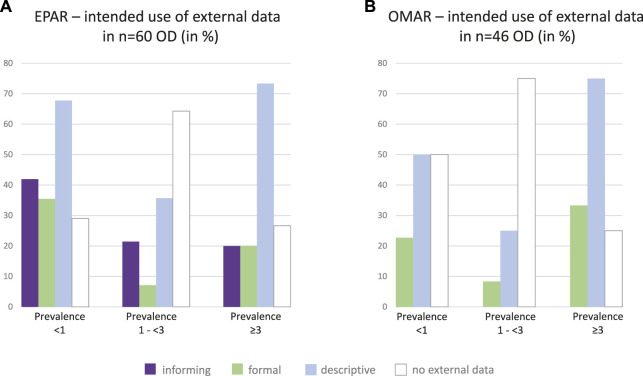
Intended use of external data. Intended use of external data in **(A)** pre-approval setting, n = 60 ODs (EPARs of all authorized products), and **(B)** “significant benefit” justification, n = 46 ODs (OMARs of all authorized products which needed to comply with the “significant benefit” criterion) were categorized and analyzed according to prevalence groups. No informing use was observed in the OMARs. EPAR: European public assessment report; OD: orphan designation; OMAR: Orphan maintenance assessment report.

The level of detail both on the external data as such and with respect to their usefulness or limitations differed between the published reports. All data had to be used descriptively to be included in our analysis (Figure 5). Only a subset was used to inform subsequent clinical development or regulatory decision-making or was formally used as an external control. The informative role was most prominent for the rarest conditions in the pre-authorization phase, while no clear trend could be identified for the formal use of external data.

Typical settings, purposes, and main criticisms of external data as described in the EPARs and OMARs are shown in Table 4. In view of the non-standardized reporting, we did not consider them suitable for quantification or prioritization. However, from the systematic review of clinical developments and subsequent regulatory assessment, we recognized the three known recurrent themes for external data: informing the subsequent trial design or decision making, contextualization (either formal or descriptive), and reassurance (descriptive). External data were generally referenced to underline the lack of efficacy of alternative treatments and thereby support the transformational benefits of the product under review rather than to accurately determine their relative effectiveness.

**TABLE 4:** Setting, purpose, and limitations of external data.

**Typical settings**	
	• Ultra-rare disease
	• Rapidly progressive disease with devastating prognosis
	• No treatment alternative apart from supportive treatment
	• Salvage therapy without agreed standard of care (late line)
	• Complex settings (surgery or transplantation as treatment alternatives)
	• Repurposing of known active substance
	• Pivotal study with a pharmacodynamic endpoint
	• More than one satisfactory method authorized for establishing SB by indirect methods
	• Demonstration of well-established use
	• Observational data used to **inform clinical study** (study population/endpoints/success criteria/non-inferiority margin)
	• **Inform** regulatory decision making (extrapolation, wording of indication)
**Collection of purposes**	• **Contextualization and reassurance** (Demonstration of unmet medical need, rule out underperformance of control arm, confirm transformational benefit; relevance of treatment effects, external control)
	• Representativity of the study population
	• Consistency/robustness of effect (especially if treatment landscape changed since study initiation or very small pivotal trials) (**reassurance**)
**Main limitations**	• High risk of bias and confounding
	• Major differences in baseline characteristics/study populations (including reporting)
	• Uncertainty with respect to comparability of diagnostic criteria
	• Differences/Uncertainty with respect to definition for efficacy criteria and collection methodology
	• Retrospective considered unsuitable in view of dynamic changes
	• Lack of quality assurance/source data verification
	• Double reporting
	• Aggregate data, only

SB: significant benefit.

External data were considered pivotal by the regulators only exceptionally. There were two MAAs for two active substances which were already established treatment options for the targeted orphan condition, but not (yet) centrally authorized in the EU. In some instances, the external data were explicitly excluded from the decision-making, while in others the weight by which they contributed to the regulatory decision-making could not be determined.

## Discussion

This study examined how external data on efficacy aspects contributed to either the clinical development or regulatory decision-making for products eligible for ODs. In the absence of a precise and universally accepted definition of RWD, we decided to address RWD in terms of administrative data and SCD, that is, categorization according to its expected depth of clinical information. We included “re-used” data from external trials (ETD) in our analysis to underline the similar aims pursued by either RWD and (typically published) ETD in regulatory submissions, especially with respect to contextualization (Tenhunen et al., 2020; Derman et al., 2022).

Our data are in line with other publications in the field but provide more granularity with respect to characteristics of the settings, purposes, and limitations of external data and focus on orphan conditions only. Our broad sample of ODs from recent regulatory decisions is largely representative of other orphan developments (European Medicines Agency, 2022b) with respect to the distribution of ATC codes, proportion of mandatory assessment of SB, or of CMA or EXC. Without unduly emphasizing the quantitative results of sometimes small subgroups, the following findings deserve more detailed discussion:

One important observation is that none of the RWD was identified as purely administrative such as claim or prescription data. This may be because very rare diseases are often grouped within ATC codes which does not allow their identification in such data (Aymé et al., 2015). More importantly, administrative data are most likely not sufficiently detailed with respect to efficacy outcomes compared to what is expected for establishing a favorable benefit/risk balance for a new treatment submitted for MA (Simon et al., 2022). Efficacy endpoints are supposed to mirror the improvement in the condition of the patient by operationalized outcome measures. The requirements for data collection for a quantification of benefit as described in respective regulatory guidelines are neither likely to be followed nor recorded in clinical practice focused on patient care (CHMP, 2016).

Furthermore, the higher proportion of MAAs with external data which were withdrawn before approval compared to successful MAs may illustrate that the submission of external data does not necessarily compensate for the lack of other convincing data for establishing a favorable benefit/risk balance of a product.

The high proportion of both ETD and SCD in MAAs for products which were approved as CMA might be explained by the fact that the unmet need or the “major therapeutic advantage” of the product under review needs to be demonstrated in order to be eligible for CMA (European Comission, 2006). This is a typical setting for the two recurrent themes for use of external data: contextualization and reassurance of the transformative nature of the product under review.

Overall, SCD were more commonly described than ETD in the EPARs reflecting the pre-authorization period and efficacy evaluation. The highest proportions of SCD were found in submissions targeting the rarest conditions, those without existing treatment alternatives, and those with SATs as pivotal trials. There is a high likelihood that clinical experience in all these areas is limited. In addition, data derived from pivotal SAT for MA may only be acceptable if sufficiently justified by e.g. reference to a lack of appropriate comparators (CHMP, 2006). In addition, clinical development may be hampered by a lack of validated efficacy endpoints. Here, external data can be used to inform on critical aspects for subsequent clinical trials with a candidate product. Longitudinal observational data often documented by experts (both retrospective and prospective data collection) were used, for example, to define clinically relevant and measurable endpoints or to shed light on the potential surrogacy of intermediate endpoints. Furthermore, external data may contribute to determine the most suitable duration of a clinical trial, or the most appropriate population to be included. Sometimes, patients included in the observational studies at one expert center had also been included in the pivotal trial of the product under review which either provided valuable baseline information on the pace of disease progression, for example, or indicated the extent of variability between patients. This approach may not be applicable across all disease types, but may still provide valuable information, for example, on sample size considerations or the need for standardization in the setting of a subsequent trial. The creation of the European Reference Network of Disease Registries on rare diseases and the associated more systematic data collection according to sub-specialties hold, therefore, the most promise for improving the efficiency of clinical trials in the future (Kölker et al., 2022).

The frequent data collection on efficacy with a real-world perspective beyond MA in the setting of very low prevalence, CMA, EXC, and for rare diseases for which the product is the first MA within the therapeutic field, further stresses the uncertainty and the continued need to consolidate the findings observed around the time of the initial MA in these circumstances. The plausibility of these findings underlines the rigor of our systematic analysis of three recent years of assessments of orphan MAAs at the EMA. Presently, information on the methods of post-approval data collection is limited. This may improve in the future if systematic publication of protocols of observational studies, including their protocols, in the ENCePP PAS registry will start, as already promoted on several occasions in events on real-world evidence hosted by the EMA (ENCePP, 2022).

Submission of external data during the pre-authorization phase was frequently seen in ODs for which RWD collection was also recommended for the post-approval phase. This underlines the importance of data collection along the entire life-cycle of some specific OMPs targeting either the rarest conditions or more complex treatment settings (such as gene therapy or transplantation in our sample). Systematic data collection on the natural history of a disease, for which a (first) drug target is discovered, may be key to making subsequent or parallel drug development and regulatory decision-making more efficient. Early interaction between drug developers, patient organizations, and registry holders is crucial for defining patient-relevant outcomes for clinical trials with a new active substance (Murphy et al., 2021). In addition, parallel consultation between regulators and HTA focusing on RWD collection may accelerate the transition from drug development to clinical practice (European Medicines Agency, 2019).

Contrary to the observation of more SCD discussed in the pre-authorization setting, more ETD were submitted for demonstrating SB in OMARs with a notable increase in submission over the last 3 years. This reflects specifically the situation of products which need to confirm orphan status in the presence of alternative treatment options. As already noted in the joint evaluation of the orphan and pediatric legislation, many orphan drug developments cluster in specific therapeutic areas. The fact that the majority of products of our broad sample of 72 ODs had to demonstrate SB in the first place, and that it is possible to reference existing clinical trials so frequently, are illustrative of the intense research activity in some rare diseases—as long as the treatment targets are known.

In Table 4, we collected typical settings, purposes, and main criticisms of external data as described in the EPARs and OMARs. We recognized the three recurrent themes for use of external data in line with other publications in the field (Franklin et al., 2019; Dagenais et al., 2022). The most influential external data on efficacy submitted during the pre-authorization phase concerned the rarest diseases and those without authorized treatment alternatives. Another prominent theme of using external data was contextualization, mainly in the setting of MAAs with a non-comprehensive data package (CMA and EXC or based on SAT) and in MAs granted in ODs without the need to demonstrate SB. All these characteristics denote therapeutic areas with the highest degree of unmet medical need. The often poor prognosis of patients suffering from devastating diseases may be one reason to accept a higher degree of uncertainty at the time of MA. In our impression, external data were then frequently used as a reassurance that the candidate product is indeed targeted to an area of unmet medical need rather than truly aiming at establishing comparative effectiveness in the pre-approval setting. In addition, it appeared that when the product under assessment was associated with compelling efficacy (i.e., the treatment effect size difference was especially large), then the lack (of information) on the quality of external data was less relevant compared to the settings in which it was important to rule out the inferiority of the product under review. Concato et al. (2020) already underlined the necessity to consider the specific context for data collection. To fully leverage the potential of RWD for regulatory decision-making, it will be crucial to define appropriate quality indicators for RWD beyond the already ongoing projects of better describing RWD and making them FAIR (Arlett et al., 2022). In our sample of reports, it was not possible to judge the quality of data collected on the product itself in the setting of an early access program (such as compassionate use). Although the inclusion of such data might be helpful to address uncertainty, especially in the setting of small clinical trials, it also needs to be stressed that compassionate use programs are primarily intended for patient care and not for evidence generation (Borysowski et al., 2017; European Medicines Agency, 2022a). Therefore, the weight of such data in the overall decision-making may still be limited even if it is mentioned in the EPARs.

One aspect which appeared more prominent for ETD than for SCD was the reporting format of the external data and whether it allowed for quality assessment. Only exceptionally individual patient-level data were referenced, although the depth of information available from, for example, other clinical trials would likely be considered beneficial for subsequent indirect comparisons. Both CHMP and COMP often criticized the negative impact of having only aggregate data reported and, therefore, being restricted to a high-level comparison across trials with relevantly different baseline characteristics. The importance of an in-depth description of patient characteristics is equally relevant for observational and interventional trial data to address confounding factors and bias (European Medicines Agency, 2021). Patients are characterized in the greatest depth in clinical trials, but much of this information may be lost for the purpose of a scientific publication (Karpen et al., 2021).

The strength of our study lies in the large number of ODs and their detailed characterization by referring to regulatory information publicly available for each MAA. The extraction of data from public reports, however, is also one of the important limitations. This review cannot comprehensively describe all the opportunities of external data in the setting of an initial MAA but only those considered worth mentioning on a case-by-case basis by the regulatory assessment team. It remains unclear in how many MAAs more external data had been submitted that was not considered sufficiently relevant by the CHMP or COMP to be discussed in the public reports. In addition, withdrawal OMARs only report on the data submitted by the applicant at the time of MAA but do not include information on the discussion on the responses to the list of questions by the COMP. Therefore, either over- or underestimation as to the use of external data in EPARs and OMARs is still possible. Furthermore, our analysis is limited to MAAs for initial licensing, although RWD might play a larger role in the extension of indications (Franklin et al., 2021; Flynn et al., 2022). Furthermore, our broad categories for the types of external data resulted in a loss of information with respect to the study design or additional quality indicators (such as prospective/retrospective data collection). In view of the difficulty of actually determining the quality of data and its assessment according to the high-level description in assessment reports, we rather focused on the characteristics of the clinical developments to identify special opportunities for external data (Concato et al., 2020).

## Conclusion

Our study reviews the use of external data in a large number of ODs spanning three years of regulatory decision-making in the setting of rare diseases at the European Medicines Agency. The high number of ODs allowed the definition of specific settings for which RWD seem to be especially promising in improving the efficiency of drug development. External data, if systematically and comprehensively collected and analyzed, can provide valuable information for both clinical development and regulatory decision-making. Agreement on the approach of data collection in the framework of scientific advice may strengthen the prospective nature of the data collection which ultimately may increase the acceptability of RWD. Ongoing initiatives of fostering data collection from patients’ everyday experiences, using common data models and complying with the FAIR principles (findable, accessible, interoperable, and re-usable) will hopefully contribute to more efficient drug development in the field of orphan conditions. Overall, our findings strengthen the impression that RWD are of particular importance in the area of rare diseases and, if properly used and of sufficient high quality, may serve as a pacemaker for the future use of RWE in drug development.

## Data Availability

The original contributions presented in the study are included in the article/Supplementary Material; further inquiries can be directed to the corresponding author.

## Abbreviations

ATC, Anatomical Therapeutic Chemical classification; CHMP, Committee for Medicinal Products for Human Use; COMP, Committee for Orphan Medicinal Products; CMA, conditional marketing authorization; EAP, early access program; EMA, European Medicines Agency; EPAR, European public assessment report; ETD, external trial data; EXC, marketing authorization under exceptional circumstances; MA(A), marketing authorization (application); MP, medicinal product; OD, orphan designation; OMAR, orphan maintenance report; OMP, orphan medicinal product; RCT, randomized controlled trial; RWD, real-world data; SAT, single-arm trial; SB, significant benefit; SCD, structured clinical data.
