# Supplementary material for: Licensing of Orphan Medicinal Products—Use of Real-World Data and Other External Data on Efficacy Aspects in Marketing Authorization Applications Concluded at the European Medicines Agency Between 2019 and 2021
Source: Front Pharmacol. 2022 Aug 11;13:920336. doi: 10.3389/fphar.2022.920336 (PMC9413272; doi:10.3389/fphar.2022.920336)
Supplement: Supplementary file 1 [file Table1.pdf]

Supplementary table S1: Contribution of external data (ETD or SCD) across different characteristics collected for the sample of ODs included in marketing authorizations of orphan designated products concluded at the EMA between 2019-2021

Subset	# of EPARs	SCD in # of EPAR	%SCD	ETD in # of EPAR	%ETD	SCD+ETD in # of EPAR	%SCD+ETD	no external data in # of EPAR	%no external data	"real world" post-MA in # of EPAR	%	# of OMAR (MA with SB)	SCD in # of OMAR	%SCD	ETD in # of OMAR	%ETD	SCD+ETD in # of OMAR	%SCD+ETD	no external data in # of OMAR	%no external data
All analysed	72	24	**33**	4	**6**	18	**25**	26	**36**	*19*	**32**	46	3	**7**	13	**28**	7	**15**	23	**50**
All MA	60	20	**33**	4	**7**	14	**23**	22	**37**											
MAA withdrawn	9	4	**44**	0	**0**	3	**33**	2	**22**	NA	**NA**	NA								
MA refused	3	0	**0**	0	**0**	1	**33**	2	**67**	NA	**NA**	NA								
**All MA**																				
**2019**	12	5	**42**	0	**0**	2	**17**	5	**42**	3	**25**	10	0	**0**	0	**0**	1	**10**	9	**90**
**2020**	23	7	**30**	2	**9**	5	**22**	9	**39**	9	**39**	20	1	**5**	5	**25**	5	**25**	9	**45**
**2021**	25	8	**32**	2	**8**	7	**28**	8	**32**	7	**28**	16	2	**13**	8	**50**	1	**6**	5	**31**
Full MA	39	13	**33**	2	**5**	5	**13**	19	**49**	7	**18**	32	3	**9**	7	**22**	3	**9**	19	**59**
CMA	17	5	**29**	2	**12**	8	**47**	2	**12**	8	**47**	12	0	**0**	5	**42**	4	**33**	3	**25**
EXC	4	2	**50**	0	**0**	1	**25**	1	**25**	4	**100**	2	0	**0**	1	**50**	0	**0**	1	**50**
Prevalence <1	**31**	15	**48**	1	**3**	6	**19**	9	**29**	*14*	**45**	**22**	1	**5**	6	**27**	4	**18**	11	**50**
Prevalence 1-<3	**14**	2	**14**	0	**0**	3	**21**	9	**64**	*1*	**7**	**12**	1	**8**	2	**17**	0	**0**	9	**75**
Prevalence >=3	**15**	3	**20**	3	**20**	5	**33**	4	**27**	4	**27**	12	1	**8**	5	**42**	3	**25**	3	**25**
New Active substance (b)	51	15	**29**	4	**8**	12	**24**	20	**39**	19	**37**	37	1	**3**	13	**35**	5	**14**	18	**49**
Known active substance	9	5	**56**	0	**0**	2	**22**	2	**22**	0	**0**	9	2	**22**	0	**0**	2	**22**	5	**56**
Pivotal study SAT (a)	**24**	10	**42**	1	**4**	9	**38**	4	**17**	12	**50**	**16**	0	**0**	5	**31**	4	**25**	7	**44**
Pivotal study RCT	**34**	10	**29**	2	**6**	4	**12**	18	**53**	6	**18**	**28**	3	**11**	6	**21**	3	**11**	16	**57**
Pivotal studies RCT+SAT	2	0	**0**	1	**50**	1	**50**	0	**0**	1	**50**	2	0	**0**	2	**100**	0	**0**	0	**0**
OD without SB	13	9	**69**	0	**0**	2	**15**	2	**15**	6	**46**	NA								
OD with SB (c)	47	11	**23**	4	**9**	12	**26**	20	**43**	13	**28**	46	3	**7**	13	**28**	7	**15**	23	**50**
OD maintained at MA	46	17	**37**	2	**4**	13	**28**	14	**30**	16	**35**	**33**	2	**6**	10	**30**	7	**21**	14	**42**
OD withdrawn at MA	14	3	**21**	2	**14**	1	**7**	8	**57**	*3*	**21**	13	1	**8**	3	**23**	0	**0**	9	**69**
ATC A	8	0	**0**	0	**0**	5	**63**	3	**38**	*3*	**38**	4	0	**0**	0	**0**	0	**0**	4	**100**
ATC B	7	1	**14**	0	**0**	1	**14**	5	**71**	*2*	**29**	7	1	**14**	0	**0**	0	**0**	6	**86**
ATC C,P,R	3	0	**0**	0	**0**	2	**67**	1	**33**	*0*	**0**	2	0	**0**	1	**50**	0	**0**	1	**50**
ATC H	3	2	**67**	0	**0**	0	**0**	1	**33**	*0*	**0**	3	2	**67**	0	**0**	1	**33**	0	**0**
ATC J	4	1	**25**	0	**0**	1	**25**	2	**50**	*2*	**50**	3	0	**0**	1	**33**	0	**0**	2	**67**
ATC L	27	3	**11**	4	**15**	9	**33**	10	**37**	*8*	**30**	22	0	**0**	9	**41**	5	**23**	8	**36**
ATC M	3	2	**67**	0	**0**	1	**33**	0	**0**	*2*	**67**	2	0	**0**	2	**100**	0	**0**	0	**0**
ATC N	5	5	**100**	0	**0**	0	**0**	0	**0**	*2*	**40**	3	0	**0**	0	**0**	1	**33**	2	**67**

ATC: anatomical therapeutic chemical classification, CMA: conditional marketing authorization, EPAR European public assessment report, ETD: external trial data, EXC: MA under exceptional cirucmstances MA: marketing authorization, OD: orphan designation, OMAR orphan maintenance report
RCT: randomized clinical trial, SAT: single-arm trial, SB: significant benefit, SCD structured clinical data
(a) One OD with retrospective pivotal data was included in the SAT analysis set.
(b) One OD was classified as new active substance by the EMA, but is known and used outside the EU.
(c) Including one withdrawn OD without OMAR, excluded for analysis in the dataset with n = 46 OMARs
